# International Health: North Korean Catastrophe

**DOI:** 10.1289/ehp.113-a26

**Published:** 2005-01

**Authors:** David J. Tenenbaum

When the Democratic People’s Republic of Korea (DPR Korea, or North Korea as it is still commonly known) makes headlines, it usually concerns the country’s nuclear ambitions. But recently the environment made the news, when the United Nations Environment Programme (UNEP) issued its *DPR Korea: State of the Environment 2003* report, which describes environmental conditions in the secretive Asian country. The report paints a grim picture of a mountainous, heavily forested country facing serious environmental challenges.

The report, which is available online at **http://www.rrcap.unep.org/reports/soe/dprksoe.cfm**, was produced by officials from 20 North Korean government and academic agencies with advice from experts at UNEP and funding from the United Nations Development Programme. In a 27 August 2004 press release announcing the first-ever nationwide report on conditions in North Korea, UNEP acknowledged “a paucity of research and data on which to base reliable environmental assessments.”

The nation is mired in a morass of intertwined environmental problems. The report says North Korea’s population is projected to grow from 23 million in 2004 to 29 million in 2020. Coal warms most houses and powers most industry. It is a major cause of severe air pollution, yet according to the report, the national goal is to quintuple coal consumption by 2020. As it is, the amount of firewood cut to meet the demand for fuel jumped from 3.0 million cubic meters per year in 1990 to 7.2 million cubic meters in 1996, causing serious deforestation.

“Soil erosion has in large part been caused by the cutting down of trees on hillsides and common land,” says Paul French, author of the 2004 book *North Korea: The Paranoid Peninsula*. “This was done to make way for extra private plots where people could grow food during the famine [which began in the late 1990s]. . . . The local people had little choice as this was an extreme survival strategy in the face of the famine and government callousness and inability to provide food.”

The government turned a blind eye, says French, and people managed to get some extra food. However, the rains, when they came, simply washed off the hillsides—because most of the nation’s forests are on slopes steeper than 20 degrees, deforestation causes erosion and flooding in the watershed. In 1995, floods cost North Korea US$15 billion in damages, and soil erosion nationwide the next year was estimated at 15 tons per hectare.

Although numerous sewage treatment plants have been built in North Korea, many households in small towns and rural areas still discharge untreated sewage into surface waters. The UNEP report attributed severe stream pollution to a “decrease in investment in environmental protection and abnormal operation of waste-water/sewage treatment plants.”

In the Taedong River, which flows through the capital, Pyongyang, the effects of these inputs are compounded by the construction of a barrier at the sea to block incoming floodwaters and by low river volume. Both of these factors have reduced the river’s natural purification capacity, concentrating contaminants near waste-water discharge points. Today, the Taedong exceeds government environmental standards and continues to deteriorate.

The report cites a number of government efforts to plant trees and conserve water, indicating that officials are aware of declining environmental conditions. However, the report avoided mention of the unique political/economic context for North Korea’s environmental conditions. Ruth Greenspan Bell, who studies Asian environmental matters for the nonprofit research group Resources for the Future, says she would assume the situation in North Korea to be the same as that in countries such as the Soviet bloc before 1989 and China today—“that environmental protection, if it exists, lacks any independent role and gets subsumed to production and full-employment goals.”

The entry for North Korea in the 2004 *CIA World Fact Book* notes that this nation, “one of the world’s most centrally planned and isolated economies,” faces desperate economic conditions. The industrial infrastructure “is nearly beyond repair as a result of years of underinvestment and spare parts shortages,” and industrial output has been declining for years. The Central Intelligence Agency estimates that massive military spending supports an army of 1 million.

Bell raises a second question about the data used in the report. “It is often important to take data from societies like North Korea—in which independent data gatherers and assessors don’t exist—with a grain of salt,” she says. “Too often people feel compelled to tell authorities what they want to hear.” Still, noted UNEP director Klaus Töpfer at the report’s launch, “By bringing together the available environmental information and identifying priority issues, the report will help strengthen monitoring and assessment, policy setting, action planning, and resourcing in DPR Korea.”

## Figures and Tables

**Figure:**
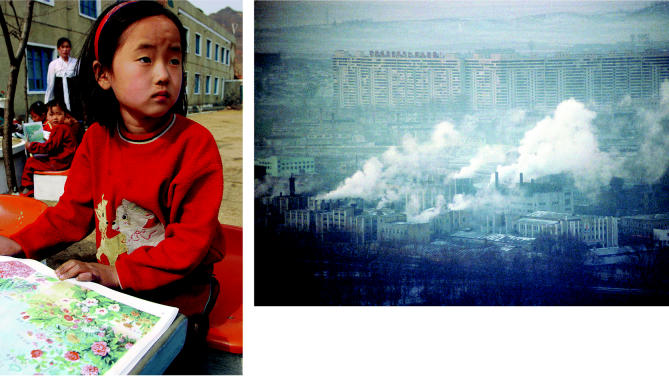
**A glimpse inside.** A dearth of data exists in the public realm on the environmental problems facing North Korea, but a new report from the United Nations Environment Programme leaves little doubt that severe pollution problems are affecting the country and its people.

